# Health Literacy and Power

**DOI:** 10.3928/24748307-20180629-01

**Published:** 2018-08-15

**Authors:** Michael K. Paasche-Orlow, Dean Schillinger, Barry D. Weiss, Timothy Bickmore, Howard Cabral, Peter Chang, Stacy C. Bailey, Darren A. Dewalt, Alicia Fernandez, Mirjam Fransen, Angela Leung, Kirsten McCaffery, Cathy D. Meade, Lauren A. McCormack, Joanne Protheroe, Ruth Parker, Russell Rothman, Don Rubin, Rima Rudd, Kristine Sørensen, Christian Von Wagner, Michael S. Wolf, H. Shonna Yin, Raymond L. Ownby

Ultimately, the goal of the field of health literacy and the purpose of *HLRP: Health Literacy Research and Practice* is empowerment—to help people gain more control over their health. Studies reported in *HLRP: Health Literacy Research and Practice* will vary, but our continuing objective is to improve individuals' and the public's health by disseminating and leveraging new discoveries and beneficial interventions related to health literacy. Articles will focus on people, families, and communities; patients, caregivers, employees, and community members; clinicians, clinics, organizations, and systems; words, numbers, and languages; mental, socioemotional processes, and interactional phenomena. We seek to understand and intervene, so people can understand better and have the ability to positively influence their health. In addition, we seek to learn and disseminate information that can catalyze a broad reshaping of public health and health care to enable institutions to work better for everyone, no matter what their level of health literacy. In this way, the goal of our work is not only patient empowerment and professional proficiency, but also disruption and transformation of the status quo to reverse institutional, systemic, and societal practices that disadvantage those with limited health literacy.

We do this because we know that health literacy is a type of power that is critical for self-determination. We also recognize (1) that the very society that generates and perpetuates limited literacy is the one that creates a discriminatory health care system, and (2) that health and illness (and health disparities) are largely determined by the maldistribution of social and environmental forces and exposures—problems that can be addressed, at least in part, through enhancing health literacy.

The fact that you can read these words means that you share in the privilege and social capital that comes with education. As a type of power, health literacy can only be strengthened with use. It often can be wielded independently of other constraints. Indeed, health literacy is not diminished when shared.

The fact that you choose to read these words means that you know that many do not share this privilege and this power. It also likely means that you want to confront the ways health is degraded and health disparities are mediated and exacerbated by low health literacy; this is a motivation that we share. Your interest in health literacy means that you will not stand idly by in the face of this wrong. How we move forward to make a difference will require a group effort and many details remain to be worked out—but our primary focus is empowerment and disruptive system transformation.

Health literacy as a field of study should challenge the conventions of a health care system that currently works best for people with the highest levels of education. However, the fact that the injustice related to limited health literacy is strongly associated with factors such as disadvantaged minority status, socioeconomic status, and nativity to name a few intensifies the ethical imperative to advance new discoveries as well as the translation and implementation of established ones. Accordingly, we have a duty to improve all aspects of our work and to innovate in measurement, participatory methods, intervention settings, cultural relevance, implementation sciences, interdisciplinary, and intersectoral work.

We must do this work in ways that include and truly engage vulnerable populations. We also must do this work with a renewed attention to privilege and power and clearly delineate the differences between health literacy and other sources of vulnerability. Adding these dimensions to our work will help assure that the work of health literacy promotes health equity and social justice. We also need to acknowledge that research about the prevalence of health literacy skills at the level of the individual and their associations with various demographic characteristics and dimensions of health status and outcomes represents only a few facets of what needs to be done. There are numerous additional dimensions of health literacy relating to communication skills of providers, organizational complexity, and resilience factors in the communities we serve—some related to health literacy practices and others unrelated to them—that should be the subject of inquiry, scholarship, and amplification. Further, we need significant advancement of interventions in all of these areas.

The field of health literacy asks all of us: What can we do with our educational privilege to make things better for those who haven't enjoyed our good fortune? How will you work to make the system better for people who do not understand or benefit from its workings? Your individual and our communal work in health literacy will represent one type of response. *HLRP: Health Literacy Research and Practice* will strive to advance your work and magnify your voice. Working together we can speak up to tear down systemic barriers that prevent people without educational privilege from fulfilling their health goals. Working together, we can better position health literacy as a powerful tool for creating personal, family, organizational, and even societal change. Our goal with this journal is to give a voice to your efforts and increase the impact of your ideas. Our work together can make a difference.

